# *N*′-(3-Eth­oxy-2-hydroxy­benzyl­idene)-3-hydroxy­naphthalene-2-carbohydrazide

**DOI:** 10.1107/S1600536808010933

**Published:** 2008-04-26

**Authors:** Jun-Tao Lei, Yan-Xia Jiang, Li-Yan Tao, Shan-Shan Huang, Hou-Li Zhang

**Affiliations:** a Department of Pharmacopedics, Jilin Medical College, Jilin 132013, People’s Republic of China; b Department of Biochemistry, Jilin Medical College, Jilin 132013, People’s Republic of China; c College of Pharmacy, Dalian Medical University, Dalian 116044, People’s Republic of China

## Abstract

In the mol­ecule of the title compound, C_20_H_18_N_2_O_4_, the dihedral angle between the benzene ring and the naphthyl ring system is 8.5 (2)°. In the crystal structure, mol­ecules are linked through inter­molecular O—H⋯O hydrogen bonds, forming chains running along the *b* axis.

## Related literature

For background on Schiff base compounds and their biological applications, see: Schiff (1864 ▶); Brückner *et al.* (2000 ▶); Harrop *et al.* (2003 ▶); Ren *et al.* (2002 ▶). For related structures, see: Diao (2007 ▶); Diao *et al.* (2007 ▶, 2008 ▶); Huang *et al.* (2007 ▶); Li *et al.* (2007 ▶). For bond-length data, see: Allen *et al.* (1987 ▶).
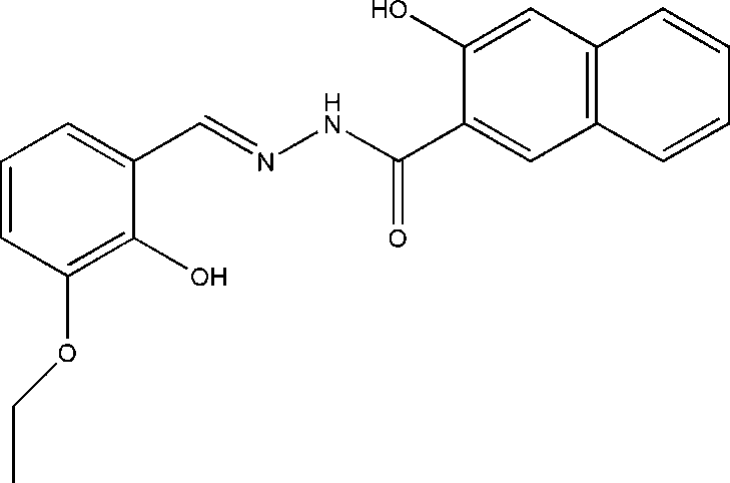


## Experimental

### 

#### Crystal data

C_20_H_18_N_2_O_4_
*M* *_r_* = 350.36
Monoclinic, 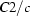

*a* = 28.420 (15) Å
*b* = 6.456 (5) Å
*c* = 18.800 (14) Å
β = 100.658 (10)°
*V* = 3390 (4) Å^3^
*Z* = 8
Mo *K*α radiation
μ = 0.10 mm^−1^
*T* = 298 (2) K
0.30 × 0.27 × 0.27 mm

#### Data collection

Bruker SMART CCD area-detector diffractometer
Absorption correction: multi-scan (*SADABS*; Bruker, 2000 ▶) *T*_min_ = 0.972, *T*_max_ = 0.974
13100 measured reflections
3503 independent reflections
2183 reflections with *I* > 2σ(*I*)
*R*_int_ = 0.057

#### Refinement

*R*[*F* ^2^ > 2σ(*F* ^2^)] = 0.060
*wR*(*F* ^2^) = 0.178
*S* = 1.06
3503 reflections
242 parameters
1 restraint
H atoms treated by a mixture of independent and constrained refinement
Δρ_max_ = 0.24 e Å^−3^
Δρ_min_ = −0.19 e Å^−3^

### 

Data collection: *SMART* (Bruker, 2000 ▶); cell refinement: *SAINT* (Bruker, 2000 ▶); data reduction: *SAINT*; program(s) used to solve structure: *SHELXTL* (Sheldrick, 2008 ▶); program(s) used to refine structure: *SHELXTL*; molecular graphics: *SHELXTL*; software used to prepare material for publication: *SHELXTL*.

## Supplementary Material

Crystal structure: contains datablocks global, I. DOI: 10.1107/S1600536808010933/pv2080sup1.cif

Structure factors: contains datablocks I. DOI: 10.1107/S1600536808010933/pv2080Isup2.hkl

Additional supplementary materials:  crystallographic information; 3D view; checkCIF report

## Figures and Tables

**Table 1:** Hydrogen-bond geometry (Å, °)

*D*—H⋯*A*	*D*—H	H⋯*A*	*D*⋯*A*	*D*—H⋯*A*
O1—H1⋯O2^i^	0.82	1.87	2.661 (3)	161
O3—H3⋯N2	0.82	1.87	2.589 (3)	146
N1—H1*A*⋯O1	0.900 (10)	1.95 (2)	2.619 (3)	130 (2)

Symmetry code: (i) 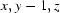.

**Fig. 1.**
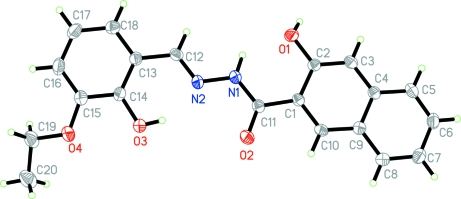
The structure of (I) with displacement parameters drawn at the 30% probability level.

**Fig. 2.**
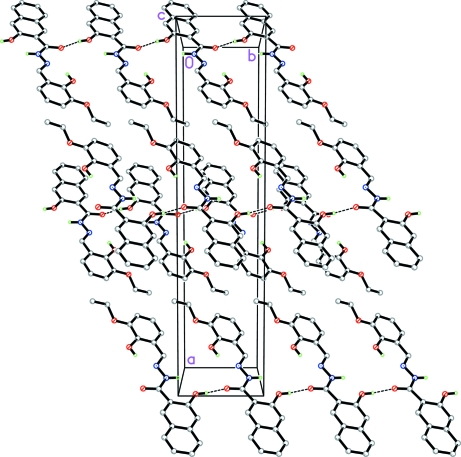
The molecular packing of (I) showing intermolecular hydrogen-bonds with dashed lines.

## supplementary crystallographic information

### Comment

The compounds derived from the condensation reactions of aldehydes with primary amines are called Schiff base compounds (Schiff, 1864). Schiff base compounds and their metal complexes have attracted much interest for their wide applications, especially for their potential pharmacological and antitumor properties (Brückner *et al.*, 2000; Harrop *et al.*, 2003; Ren *et al.*, 2002). In this paper, the preparation and crystal structure of the title compound, (I), is reported.

In the structure of (I) (Fig. 1), the naphthyl ring and 2-hydroxyphenyl methylidene hydrazide moiety are nearly coplanar with the dihedral angle between the phenyl ring and the naphthyl ring is 8.5 (2) °; the torsion angles C13—C12—N2—N1 and N2—N1—C11—C1 are 3.5 (2) and 1.4 (2)°, respectively. The methoxy group is slightly twisted out of the plane of the phenyl ring with torsion angle C15—O4—C19—C20 being 13.1 (2)°. The molecules of (I) are linked through intermolecular O–H···O hydrogen bonds, forming chains running along the *b* axis. The structure is further stabilized by intramolecular interactions N1—H1A···O1 and O3—H3···N2 (Table 1). All the bond lengths are within normal ranges (Allen *et al.*, 1987) and comparable to the values observed in other similar compounds (Diao *et al.*, 2008; Diao *et al.*, 2007; Diao, 2007; Li *et al.*, 2007; Huang *et al.*, 2007).

### Experimental

3-Ethoxysalicylaldehyde (0.1 mmol, 16.6 mg) and 3-hydroxynaphthalene-2-carboxylic acid hydrazide (0.1 mmol, 20.2 mg) were dissolved in a methanol solution (20 ml). The mixture was stirred at reflux for 1 h and cooled to room temperature. After keeping the solution in air for a few days, colorless block-like crystals were formed.

### Refinement

H1A was located from a difference Fourier map and refined isotropically. The rest of the H atoms were placed in calculated positions and constrained to ride on their parent atoms, with C–H distances of 0.93–0.97 Å, O–H distances of 0.82 Å, and with *U*_iso_(H) = 1.2*U*_eq_(C) and 1.5*U*_eq_(O and methyl C).

### Crystal data

**Table:**

C_20_H_18_N_2_O_4_	*F*_000_ = 1472
*M**_r_* = 350.36	*D*_x_ = 1.373 Mg m^−^^3^
Monoclinic, *C*2/*c*	Mo *K*α radiation λ = 0.71073 Å
Hall symbol: -C 2yc	Cell parameters from 1359 reflections
*a* = 28.420 (15) Å	θ = 2.2–24.3º
*b* = 6.456 (5) Å	µ = 0.10 mm^−^^1^
*c* = 18.800 (14) Å	*T* = 298 (2) K
β = 100.658 (10)º	Block, colorless
*V* = 3390 (4) Å^3^	0.30 × 0.27 × 0.27 mm
*Z* = 8	

### Data collection

**Table:**

Bruker SMART CCD area-detector diffractometer	3503 independent reflections
Radiation source: fine-focus sealed tube	2183 reflections with *I* > 2σ(*I*)
Monochromator: graphite	*R*_int_ = 0.057
*T* = 298(2) K	θ_max_ = 26.5º
ω scans	θ_min_ = 1.5º
Absorption correction: multi-scan(SADABS; Bruker, 2000)	*h* = −35→35
*T*_min_ = 0.972, *T*_max_ = 0.974	*k* = −8→8
13100 measured reflections	*l* = −23→22

### Refinement

**Table:**

Refinement on *F*^2^	Secondary atom site location: difference Fourier map
Least-squares matrix: full	Hydrogen site location: inferred from neighbouring sites
*R*[*F*^2^ > 2σ(*F*^2^)] = 0.060	H atoms treated by a mixture of independent and constrained refinement
*wR*(*F*^2^) = 0.178	*w* = 1/[σ^2^(*F*_o_^2^) + 0.1909*P*] where *P* = (*F*_o_^2^ + 2*F*_c_^2^)/3
*S* = 1.06	(Δ/σ)_max_ < 0.001
3503 reflections	Δρ_max_ = 0.24 e Å^−^^3^
242 parameters	Δρ_min_ = −0.19 e Å^−^^3^
1 restraint	Extinction correction: SHELXTL (Sheldrick, 2008), Fc^*^=kFc[1+0.001xFc^2^λ^3^/sin(2θ)]^-1/4^
Primary atom site location: structure-invariant direct methods	Extinction coefficient: 0.0024 (6)

### Special details

**Table:**

Geometry. All e.s.d.'s (except the e.s.d. in the dihedral angle between two l.s. planes) are estimated using the full covariance matrix. The cell e.s.d.'s are taken into account individually in the estimation of e.s.d.'s in distances, angles and torsion angles; correlations between e.s.d.'s in cell parameters are only used when they are defined by crystal symmetry. An approximate (isotropic) treatment of cell e.s.d.'s is used for estimating e.s.d.'s involving l.s. planes.
Refinement. Refinement of *F*^2^ against ALL reflections. The weighted *R*-factor *wR* and goodness of fit *S* are based on *F*^2^, conventional *R*-factors *R* are based on *F*, with *F* set to zero for negative *F*^2^. The threshold expression of *F*^2^ > σ(*F*^2^) is used only for calculating *R*-factors(gt) *etc*. and is not relevant to the choice of reflections for refinement. *R*-factors based on *F*^2^ are statistically about twice as large as those based on *F*, and *R*- factors based on ALL data will be even larger.

### Fractional atomic coordinates and isotropic or equivalent isotropic displacement parameters (Å^2^)

**Table:**

	*x*	*y*	*z*	*U*_iso_*/*U*_eq_	
O1	1.00934 (6)	−0.1963 (2)	0.34159 (8)	0.0489 (5)	
H1	1.0059	−0.3211	0.3470	0.073*	
O2	0.98937 (6)	0.4267 (2)	0.38501 (9)	0.0528 (5)	
O3	0.88269 (7)	0.6149 (3)	0.24267 (9)	0.0611 (6)	
H3	0.9039	0.5398	0.2644	0.092*	
O4	0.81068 (6)	0.8088 (3)	0.16639 (10)	0.0592 (5)	
N1	0.96591 (7)	0.1590 (3)	0.31039 (10)	0.0396 (5)	
N2	0.93488 (7)	0.2841 (3)	0.26466 (10)	0.0394 (5)	
C1	1.02434 (7)	0.1001 (3)	0.41996 (11)	0.0336 (5)	
C2	1.03361 (8)	−0.1122 (3)	0.40475 (11)	0.0363 (5)	
C3	1.06657 (8)	−0.2231 (3)	0.45120 (13)	0.0405 (6)	
H3A	1.0734	−0.3580	0.4390	0.049*	
C4	1.09062 (8)	−0.1396 (3)	0.51714 (12)	0.0377 (5)	
C5	1.12406 (8)	−0.2534 (4)	0.56714 (14)	0.0467 (6)	
H5	1.1314	−0.3888	0.5563	0.056*	
C6	1.14561 (9)	−0.1678 (4)	0.63087 (14)	0.0502 (6)	
H6	1.1670	−0.2465	0.6635	0.060*	
C7	1.13609 (8)	0.0384 (4)	0.64834 (13)	0.0498 (7)	
H7	1.1516	0.0960	0.6917	0.060*	
C8	1.10409 (8)	0.1528 (4)	0.60139 (12)	0.0431 (6)	
H8	1.0976	0.2882	0.6133	0.052*	
C9	1.08064 (8)	0.0685 (3)	0.53476 (11)	0.0364 (5)	
C10	1.04759 (8)	0.1814 (3)	0.48433 (12)	0.0372 (5)	
H10	1.0412	0.3179	0.4952	0.045*	
C11	0.99190 (8)	0.2416 (3)	0.37086 (12)	0.0365 (5)	
C12	0.91016 (8)	0.2028 (4)	0.20827 (12)	0.0402 (6)	
H12	0.9147	0.0643	0.1977	0.048*	
C13	0.87471 (8)	0.3253 (4)	0.16007 (12)	0.0392 (6)	
C14	0.86145 (8)	0.5211 (4)	0.18082 (12)	0.0426 (6)	
C15	0.82336 (9)	0.6255 (4)	0.13816 (13)	0.0471 (6)	
C16	0.80210 (9)	0.5426 (4)	0.07255 (13)	0.0562 (7)	
H16	0.7776	0.6148	0.0431	0.067*	
C17	0.81680 (9)	0.3529 (4)	0.04985 (14)	0.0566 (7)	
H17	0.8027	0.3001	0.0049	0.068*	
C18	0.85201 (8)	0.2431 (4)	0.09359 (13)	0.0465 (6)	
H18	0.8609	0.1134	0.0791	0.056*	
C19	0.76647 (9)	0.9005 (4)	0.13279 (16)	0.0612 (8)	
H19A	0.7413	0.7971	0.1251	0.073*	
H19B	0.7694	0.9590	0.0863	0.073*	
C20	0.75477 (11)	1.0676 (4)	0.18230 (17)	0.0764 (9)	
H20A	0.7498	1.0067	0.2269	0.115*	
H20B	0.7262	1.1384	0.1597	0.115*	
H20C	0.7808	1.1643	0.1920	0.115*	
H1A	0.9661 (10)	0.0234 (18)	0.2992 (15)	0.080*	

### Atomic displacement parameters (Å^2^)

**Table:**

	*U*^11^	*U*^22^	*U*^33^	*U*^12^	*U*^13^	*U*^23^
O1	0.0656 (11)	0.0304 (9)	0.0453 (10)	0.0015 (8)	−0.0038 (8)	−0.0026 (7)
O2	0.0653 (12)	0.0272 (9)	0.0598 (11)	0.0042 (8)	−0.0043 (9)	0.0031 (8)
O3	0.0697 (13)	0.0505 (11)	0.0525 (11)	0.0209 (9)	−0.0165 (9)	−0.0120 (9)
O4	0.0558 (11)	0.0509 (11)	0.0628 (12)	0.0186 (8)	−0.0098 (9)	−0.0038 (9)
N1	0.0435 (11)	0.0338 (10)	0.0395 (11)	0.0055 (9)	0.0022 (9)	0.0036 (9)
N2	0.0412 (11)	0.0362 (11)	0.0388 (11)	0.0058 (8)	0.0020 (9)	0.0052 (9)
C1	0.0351 (12)	0.0299 (12)	0.0364 (12)	−0.0008 (9)	0.0082 (10)	0.0037 (9)
C2	0.0409 (13)	0.0313 (12)	0.0365 (12)	−0.0030 (10)	0.0066 (10)	0.0005 (10)
C3	0.0442 (14)	0.0308 (12)	0.0473 (14)	0.0021 (10)	0.0105 (11)	0.0017 (10)
C4	0.0351 (12)	0.0369 (13)	0.0422 (13)	0.0013 (10)	0.0099 (10)	0.0020 (10)
C5	0.0451 (14)	0.0446 (14)	0.0514 (15)	0.0114 (11)	0.0114 (12)	0.0047 (12)
C6	0.0422 (14)	0.0616 (17)	0.0448 (15)	0.0093 (12)	0.0029 (12)	0.0051 (12)
C7	0.0435 (15)	0.0634 (18)	0.0410 (14)	−0.0001 (12)	0.0041 (12)	−0.0010 (12)
C8	0.0463 (14)	0.0407 (13)	0.0430 (14)	−0.0013 (11)	0.0098 (11)	−0.0030 (11)
C9	0.0369 (12)	0.0394 (13)	0.0342 (12)	−0.0012 (10)	0.0099 (10)	0.0025 (10)
C10	0.0442 (13)	0.0275 (11)	0.0413 (13)	−0.0002 (10)	0.0113 (11)	0.0009 (10)
C11	0.0379 (12)	0.0312 (13)	0.0407 (13)	−0.0006 (10)	0.0077 (10)	0.0045 (10)
C12	0.0438 (14)	0.0388 (13)	0.0395 (13)	0.0073 (10)	0.0118 (11)	−0.0009 (10)
C13	0.0394 (13)	0.0432 (13)	0.0357 (13)	0.0042 (10)	0.0086 (10)	0.0016 (10)
C14	0.0427 (14)	0.0478 (15)	0.0349 (12)	0.0048 (11)	0.0009 (11)	0.0013 (11)
C15	0.0449 (14)	0.0476 (15)	0.0469 (14)	0.0064 (12)	0.0032 (12)	0.0020 (12)
C16	0.0531 (17)	0.0645 (18)	0.0455 (15)	0.0097 (13)	−0.0052 (12)	0.0103 (13)
C17	0.0573 (17)	0.0694 (19)	0.0389 (14)	0.0010 (14)	−0.0023 (12)	−0.0065 (13)
C18	0.0496 (15)	0.0490 (15)	0.0418 (14)	0.0010 (12)	0.0106 (12)	−0.0036 (11)
C19	0.0419 (15)	0.0561 (17)	0.081 (2)	0.0104 (12)	−0.0009 (14)	0.0025 (15)
C20	0.0619 (19)	0.066 (2)	0.101 (2)	0.0174 (15)	0.0127 (18)	−0.0019 (18)

### Geometric parameters (Å, °)

**Table:**

O1—C2	1.370 (3)	C7—C8	1.362 (3)
O1—H1	0.8200	C7—H7	0.9300
O2—C11	1.230 (3)	C8—C9	1.414 (3)
O3—C14	1.350 (3)	C8—H8	0.9300
O3—H3	0.8200	C9—C10	1.408 (3)
O4—C15	1.372 (3)	C10—H10	0.9300
O4—C19	1.426 (3)	C12—C13	1.457 (3)
N1—C11	1.346 (3)	C12—H12	0.9300
N1—N2	1.374 (2)	C13—C14	1.395 (3)
N1—H1A	0.900 (10)	C13—C18	1.401 (3)
N2—C12	1.272 (3)	C14—C15	1.396 (3)
C1—C10	1.371 (3)	C15—C16	1.377 (3)
C1—C2	1.434 (3)	C16—C17	1.387 (4)
C1—C11	1.490 (3)	C16—H16	0.9300
C2—C3	1.360 (3)	C17—C18	1.369 (3)
C3—C4	1.407 (3)	C17—H17	0.9300
C3—H3A	0.9300	C18—H18	0.9300
C4—C5	1.413 (3)	C19—C20	1.501 (4)
C4—C9	1.425 (3)	C19—H19A	0.9700
C5—C6	1.358 (3)	C19—H19B	0.9700
C5—H5	0.9300	C20—H20A	0.9600
C6—C7	1.409 (4)	C20—H20B	0.9600
C6—H6	0.9300	C20—H20C	0.9600
			
C2—O1—H1	109.5	O2—C11—N1	121.5 (2)
C14—O3—H3	109.5	O2—C11—C1	121.1 (2)
C15—O4—C19	117.36 (19)	N1—C11—C1	117.43 (19)
C11—N1—N2	118.96 (19)	N2—C12—C13	120.4 (2)
C11—N1—H1A	123.8 (18)	N2—C12—H12	119.8
N2—N1—H1A	117.1 (18)	C13—C12—H12	119.8
C12—N2—N1	118.05 (19)	C14—C13—C18	119.3 (2)
C10—C1—C2	117.73 (19)	C14—C13—C12	120.6 (2)
C10—C1—C11	117.0 (2)	C18—C13—C12	120.0 (2)
C2—C1—C11	125.3 (2)	O3—C14—C13	123.1 (2)
C3—C2—O1	121.6 (2)	O3—C14—C15	117.0 (2)
C3—C2—C1	120.3 (2)	C13—C14—C15	119.8 (2)
O1—C2—C1	118.02 (19)	O4—C15—C16	125.3 (2)
C2—C3—C4	121.9 (2)	O4—C15—C14	115.2 (2)
C2—C3—H3A	119.0	C16—C15—C14	119.5 (2)
C4—C3—H3A	119.0	C15—C16—C17	120.8 (2)
C3—C4—C5	123.0 (2)	C15—C16—H16	119.6
C3—C4—C9	118.7 (2)	C17—C16—H16	119.6
C5—C4—C9	118.3 (2)	C18—C17—C16	120.0 (2)
C6—C5—C4	120.9 (2)	C18—C17—H17	120.0
C6—C5—H5	119.6	C16—C17—H17	120.0
C4—C5—H5	119.6	C17—C18—C13	120.3 (2)
C5—C6—C7	121.0 (2)	C17—C18—H18	119.8
C5—C6—H6	119.5	C13—C18—H18	119.8
C7—C6—H6	119.5	O4—C19—C20	107.5 (2)
C8—C7—C6	119.7 (2)	O4—C19—H19A	110.2
C8—C7—H7	120.2	C20—C19—H19A	110.2
C6—C7—H7	120.2	O4—C19—H19B	110.2
C7—C8—C9	120.9 (2)	C20—C19—H19B	110.2
C7—C8—H8	119.5	H19A—C19—H19B	108.5
C9—C8—H8	119.5	C19—C20—H20A	109.5
C10—C9—C8	122.9 (2)	C19—C20—H20B	109.5
C10—C9—C4	117.9 (2)	H20A—C20—H20B	109.5
C8—C9—C4	119.2 (2)	C19—C20—H20C	109.5
C1—C10—C9	123.3 (2)	H20A—C20—H20C	109.5
C1—C10—H10	118.4	H20B—C20—H20C	109.5
C9—C10—H10	118.4		

### Hydrogen-bond geometry (Å, °)

**Table:**

*D*—H···*A*	*D*—H	H···*A*	*D*···*A*	*D*—H···*A*
O1—H1···O2^i^	0.82	1.87	2.661 (3)	161
O3—H3···N2	0.82	1.87	2.589 (3)	146
N1—H1A···O1	0.900 (10)	1.95 (2)	2.619 (3)	130 (2)

Symmetry codes: (i) *x*, *y*−1, *z*.
